# Integrated nonlinear optical imaging microscope for on-axis crystal detection and centering at a synchrotron beamline

**DOI:** 10.1107/S0909049513007942

**Published:** 2013-05-03

**Authors:** Jeremy T. Madden, Scott J. Toth, Christopher M. Dettmar, Justin A. Newman, Robert A. Oglesbee, Hartmut G. Hedderich, R. Michael Everly, Michael Becker, Judith A. Ronau, Susan K. Buchanan, Vadim Cherezov, Marie E. Morrow, Shenglan Xu, Dale Ferguson, Oleg Makarov, Chittaranjan Das, Robert Fischetti, Garth J. Simpson

**Affiliations:** a Department of Chemistry, Purdue University, 560 Oval Drive, West Lafayette, IN 47906, USA; b GM/CA@APS, Advanced Photon Source, Argonne National Laboratory, 9700 South Cass Avenue, Argonne, IL 60439, USA; c NIDDK, National Institutes of Health, Building 50, Room 4503, 50 South Drive, Bethesda, MD 20814, USA; d Department of Molecular Biology, The Scripps Research Institute, 10550 North Torrey Pines Road, La Jolla, CA 92037, USA

**Keywords:** XRD, NLO, SHG, SONICC, centering, protein, TPE-UVF, microscopy, LCP, two-photon

## Abstract

Nonlinear optical (NLO) instrumentation has been integrated with synchrotron X-ray diffraction (XRD) for combined single-platform analysis, initially targeting applications for automated crystal centering. Second-harmonic-generation microscopy and two-photon-excited ultraviolet fluorescence microscopy were evaluated for crystal detection and assessed by X-ray raster scanning. Two optical designs were constructed and characterized; one positioned downstream of the sample and one integrated into the upstream optical path of the diffractometer. Both instruments enabled protein crystal identification with integration times between 80 and 150 µs per pixel, representing a ∼10^3^–10^4^-fold reduction in the per-pixel exposure time relative to X-ray raster scanning. Quantitative centering and analysis of phenylalanine hydroxylase from *Chromobacterium violaceum* cPAH, *Trichinella spiralis* deubiquitinating enzyme TsUCH37, human κ-opioid receptor complex kOR-T4L produced in lipidic cubic phase (LCP), intimin prepared in LCP, and α-cellulose samples were performed by collecting multiple NLO images. The crystalline samples were characterized by single-crystal diffraction patterns, while α-cellulose was characterized by fiber diffraction. Good agreement was observed between the sample positions identified by NLO and XRD raster measurements for all samples studied.

## Abstract 2

Nonlinear optical (NLO) instrumentation has been integrated with synchrotron X-ray diffraction for combined single-platform analysis, examining the viability of NLO microscopy as an alternative to the conventional X-ray raster scan for the purposes of sample centering. Second-harmonic generation microscopy and two-photon excited ultraviolet fluorescence microscopy were evaluated for crystal detection, and assessed by X-ray raster scanning.

## 1. Introduction   

The high photon flux and energy tunability of synchrotron radiation sources have made them indispensable tools for X-ray analysis, with applications spanning protein structure determination through materials science and nanotechnology (Rasmussen *et al.*, 2011 ▶; Moukhametzianov *et al.*, 2008 ▶; Bates *et al.*, 2006 ▶; Berger *et al.*, 2010 ▶; Dauter, 2006 ▶; Ihee *et al.*, 2010 ▶; le Maire *et al.*, 2011 ▶; Parker *et al.*, 2006 ▶; Riekel *et al.*, 2005 ▶). The increasing drive toward tighter focusing has enabled structure determination on ever-smaller crystals and sub-domains within materials, but presents growing challenges for reliable crystal centering. These challenges are particularly relevant for protein crystal diffraction, in which the drive toward fully automated X-ray diffraction analysis at synchrotron sources has introduced bottlenecks in sample positioning (Andrey *et al.*, 2004 ▶; Moukhametzianov *et al.*, 2008 ▶; Pothineni *et al.*, 2006 ▶; Aishima *et al.*, 2010 ▶; Cherezov *et al.*, 2009 ▶; Stepanov *et al.*, 2011*a* ▶). Diffraction-quality protein crystals are typically obtained through crystallization screenings, followed by optimization, and then are placed into cryo-loops, which are flash-cooled in liquid nitrogen to reduce X-ray damage and aid in sample handling (Dobrianov *et al.*, 1999 ▶; Karain *et al.*, 2002 ▶). High-throughput methods for automated crystal positioning are frustrated by complications of reliable centering of smaller and smaller protein crystals within more complex and turbid matrices. The current most reliable methods for crystal centering involve rastering the sample using a focused X-ray beam (Accardo *et al.*, 2010 ▶; Hilgart *et al.*, 2011 ▶; Cherezov *et al.*, 2009 ▶; Stepanov *et al.*, 2011*a* ▶; Aishima *et al.*, 2010 ▶; Song *et al.*, 2007 ▶). From the resulting X-ray diffraction images recorded as a function of sample position in the beam, protein crystals are centered based on the locations of strongest Bragg-like diffraction. X-ray fluorescence raster is also relatively fast, but it requires a convenient X-ray fluorescent element to be present in the crystal (Stepanov *et al.*, 2011*a* ▶).

While generally successful, X-ray raster scanning suffers from several limitations. First, the method is relatively slow, often utilizing >2 s per pixel (raster cell), corresponding to analysis times from several minutes up to an hour depending on the number of cells in the raster grid and on the exposure time (Aishima *et al.*, 2010 ▶). Rastering is commonly performed first with a coarse grid, and then a finer grid, to minimize the number of cells, and to increase speed. The total pixel number is in turn dependent on the size of the X-ray beam, the speed of the detector and analysis, as well as the scanned size of the cryo-loop and the crystal itself (Cherezov *et al.*, 2009 ▶; Song *et al.*, 2007 ▶). Recent advances in diffraction image read times using single-photon-counting arrays (pixel array detectors) (Broennimann *et al.*, 2006 ▶), allowing integration times as low as 2 ms per image (Aishima *et al.*, 2010 ▶), can significantly reduce the time frame for raster scanning measurements. However, the time required for raster scanning will still ultimately be limited by the collective times required to obtain sufficient signal to noise (S/N) in a given pixel, to translate the sample through the X-ray source, and to reconstruct the crystal positions based on automated analysis of the compiled diffraction images. Diffraction is a relatively inefficient process with far more X-ray photons absorbed or inelastically scattered than detected for diffraction analysis, contributing to sample damage, even under the cryogenic conditions typically utilized. With small crystals or beams, incident X-ray intensities must be increased accordingly to achieve diffracted intensities equivalent to those for large crystals, thereby increasing absorbed X-ray dose and exacerbating damage. Alternative methods for automated loop centering based on optical imaging include bright-field image analysis and ultraviolet fluorescence (UVF) microscopy, which takes advantage of intrinsic fluorescent properties of protein crystals (Jain & Stojanoff, 2007 ▶; Vernede *et al.*, 2006 ▶; Pohl *et al.*, 2004 ▶; Andrey *et al.*, 2004 ▶; Pothineni *et al.*, 2006 ▶). However, algorithms for protein crystal centering (*e.g.* based on crystal edge-finding algorithms) are error-prone for microcrystals and turbid matrices, such as lipidic cubic phase (LCP). Methods optimized for analysis within the mother liquor often prove unreliable for a loop-mounted crystal, in part because algorithms often cannot easily distinguish between the loop, features in the cryo-cooled mother-liquor and the crystal. Furthermore, both bright-field and UVF imaging are challenging to reliably implement in turbid matrices, where optical scattering frustrates reliable crystal imaging. UVF also has a potential disadvantage of inducing UV photodamage to samples from long exposures, or in highly labile proteins, but the exposure times required for imaging are typically short enough to minimize such effects (Vernede *et al.*, 2006 ▶; Chen *et al.*, 2009 ▶; Nanao & Ravelli, 2006 ▶).

More recently, nonlinear optical imaging (NLO) methods such as second-harmonic generation (SHG) and two-photon-excited UV fluorescence (TPE-UVF) have emerged as viable alternatives for high-contrast crystal visualization (Kissick *et al.*, 2010 ▶; Madden *et al.*, 2011 ▶). SHG, or the frequency doubling of light, is symmetry forbidden in disordered media (*e.g.* amorphous protein aggregates or proteins in solution) but is allowed for certain classes of crystals (Haupert & Simpson, 2011 ▶). Fortuitously, the chirality intrinsic to proteins typically results in the adoption of SHG-active crystal classes. Recent quantum chemical calculations suggest an SHG coverage of approximately 84% of protein crystals in the Protein Crystal Database using an optimized instrument (Haupert *et al.*, 2012 ▶). TPE-UVF provides a complimentary method to SHG for protein crystal detection, with contrast dependent on the presence of aromatic side-chains (primarily tryptophan), independent of crystallinity. Crystals that are weakly active to SHG imaging but contain fluorescent amino acid residues can be detected (Madden *et al.*, 2011 ▶). Furthermore, TPE-UVF can aid in distinguishing SHG-active small-molecule and salt crystals from protein crystals.

The high selectivity for crystals and negligible background from disordered protein aggregates typically produces high-contrast SHG images, which are highly compatible with automated image analysis algorithms designed for protein crystal detection and centering (Haupert & Simpson, 2011 ▶). SHG measurements have recently enabled crystal detection for diffraction centering using off-line instrumentation (Kissick *et al.*, 2013 ▶), in which protein crystals were first imaged under cryogenic conditions with an SHG microscope, and then manually compared with diffraction images obtained by X-ray raster scanning with good agreement. A major benefit of NLO instruments is the reduction in time required to determine crystal locations with high contrast, as measurements for an entire loop can be obtained in as little as a few seconds, compared with tens of minutes routinely required for X-ray raster imaging. The spatial resolution of NLO instruments is also high (∼1–2 µm), whereas X-ray diffraction (XRD) rastering with this type of resolution would take substantially longer to scan an area equivalent to that of the entire NLO image (>72 h at 1 s per pixel for a 512 × 512 pixel image). Furthermore, reducing the reliance on X-ray raster imaging would minimize X-ray-induced sample damage (Hilgart *et al.*, 2011 ▶; Ravelli & Garman, 2006 ▶).

By integrating SHG and TPE-UVF imaging directly into a synchrotron X-ray diffraction beamline, the robotic controls, automated positioning capabilities, cryogenics and other beamline utilities of high-throughput synchrotron facilities can be leveraged. However, the spatial constraints of a typical synchrotron X-ray experimental hutch represent a nontrivial hurdle for development of compatible NLO instrumentation. Typical research NLO instruments occupy a large footprint (an optical table approximately 120 cm × 300 cm), far greater than the space available on a typical beamline. In this work, two complementary prototypes for an on-line compatible instrument combining synchrotron XRD and NLO imaging are described. Assessment of these systems was performed by direct comparisons between NLO images and those obtained by X-ray diffraction rastering.

## 2. Experimental methods   

**Figure 1:**
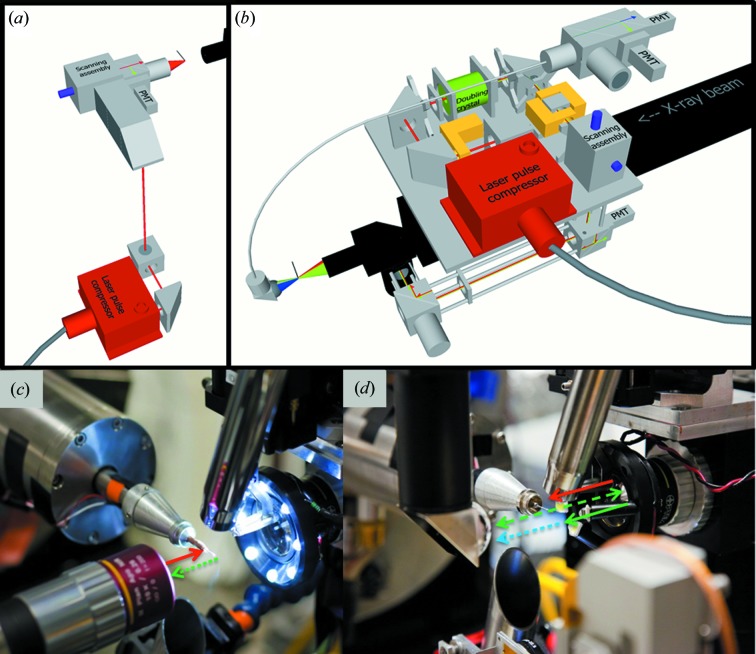
(*a*) Schematic of the downstream NLO microscope; (*b*) schematic of the upstream NLO microscope; (*c*) close-up view of the downstream NLO microscope, with the solid arrow representing incident laser propagation (red, 1060 nm) and dashed arrows representing the frequency-doubled signal (green, SHG at 530 nm); (*d*) close-up view of the upstream NLO microscope, with solid arrows representing incident laser propagation (red, 1060 nm; green, 530 nm) and dashed arrows representing the measured signal (green, SHG at 530 nm; blue, TPE-UVF).

Two separate instruments were designed and constructed for integrating XRD and NLO imaging, each with its own advantages and limitations. The upstream version introduced the incident light coaxial and parallel with the direction of the X-ray beam path, while the downstream system was coaxial and anti-parallel. The upstream version was designed to fully integrate with the existing optical path, while the downstream version was optimized for high flexibility and compatibility with diverse beamline configurations. Both systems were rated as Class I laser systems on-site, with enclosed beam paths, shutters and interlocks to ensure no exposed collimated optical radiation. The integrated NLO microscopes were installed at beamlines 23-ID-B and 23-ID-D at the Advanced Photon Source (APS) at Argonne National Laboratory in Argonne, IL, USA. A basic schematic of the instruments and beam paths as they were installed on the synchrotron beamline can be seen in Fig. 1 ▶. Detailed descriptions and photographs are provided.

### 2.1. Integrated nonlinear optical microscope designs   

The upstream illumination NLO system was designed to sit above the existing instrumentation at GM/CA beamline 23-ID-B at the APS, and couple directly into the existing optical path. A Fianium FemtoPower 1060 ultrafast fiber laser was utilized, producing ∼160 fs pulses centered around 1060 nm, with a 50 MHz repetition rate, maximum power of 1.5 W, allowing for a maximum power of ∼140 mW at the sample, with 80% of the overall loss arising from the objective. The Fianium source was composed of an oscillator coupled *via* a 1.5 m fiber to a dispersion compensator and free-space coupler unit, with dimensions of approximately 15 cm × 13 cm × 8 cm. A heated doubling crystal (Newlight Photonics Inc., SHG1663-IM, HTS 85141000) was permanently assembled in the beam path, with the fundamental beam focused into the crystal with a plano-convex lens (*f* = 35 mm) and collimated with another plano-convex lens (*f* = 100 mm) after the doubling crystal. The efficiency of SHG from the doubling crystal was controlled by either introducing or removing a 1064 nm zero-order half-wave plate using a flip mount (New Focus, 8892-K). The scanning assembly consisted of a galvanometer mirror (Cambridge Technology, 6210H) and resonant scanning mirror (Cambridge Technology, 1-003-3002509), controlling the beam position on the horizontal slow-scan and vertical fast-scan axes, respectively. The beam was directed into a telocentric lens pair consisting of two plano-convex lenses (*f* = 75 mm and *f* = 250 mm) leading to an additional 3.3× beam expansion after the scan head. The incident light then reflected off a dichroic mirror stack (Semrock, PBP01-529/23-25x36 and Chroma, 900dcsp) designed to reflect 1060 nm and s-polarized 530 nm incident light. The p-polarized component of the returning 530 nm light was transmitted by this same dichroic for epi-detected SHG (*i.e.* SHG detected in the backward direction through the same objective as the incident light). High-reflectivity dichroic mirrors for both 1060 nm and 530 nm light (Semrock, FF550-Di01-25x36) delivered both wavelengths to the back aperture of the 10× objective (Optem, 28-21-10), which was modified with a ∼1.2 mm hole bored through the center to allow X-ray access. In epi, the p-polarized SHG returning through the dichroic mirror was passed through a bandpass filter set (Chroma, HQ530/30m and CVI, 03FCG567/KG3) and into a compact photomultiplier tube (PMT) module (Hamamatsu, H10722-10). SHG and TPE-UVF were collected in the transmission direction by a plano-convex lens (*f* = 25.4 mm) affixed to a right-angle prism using optical epoxy (Norland Optical Adhesive 63). Another plano-convex lens (*f* = 25.4 mm) coupled the detected light into a near-UV-compatible liquid light guide (Oriel Instruments, 77554) collimated with a plano-convex lens (*f* = 25.4 mm) into the detection assembly. Both the SHG and TPE-UVF were then reflected off a primary dichroic beam splitter (Semrock, FF555-Di03-25x36), then separated at a second dichroic beam splitter (Chroma, z1064rdc-sp) for selective detection of SHG (through Chroma, HQ530/30m and CVI, 03FCG567/KG3 filters) and TPE-UVF (through Semrock, SP01-532RS-25 and FF01-440/SP-25 filters). Both the SHG and TPE-UVF were focused onto the faces of the PMT modules (Hamamatsu, H10722-10) by a plano-convex lens (*f* = 60 mm) positioned between the primary and secondary dichroic beam splitters. Backlight illumination was achieved using an LED (ThorLabs, MCWHL2) passing through the primary dichroic beam splitter and into the liquid light guide. The illumination light was then focused through the trans-SHG/TPE-UVF collection optics and onto the sample.

The downstream NLO system was also designed with the optical axis of the objective co-axial with the axis of X-ray propagation [Figs. 1(*a*) and 1(*b*) ▶], using a similar laser source. The size constraints associated with this beamline, specifically the restrictions imposed by the support structure of the beamline and the area and instruments surrounding the sample, limited the available footprint of the NLO system to 39 cm × 19 cm. The scanning assembly was composed of dual galvanometers (Cambridge Technologies, 6210HSM40B), mounted in a two-dimensional galvo 30 mm cage cube (Thorlabs, GCM002), with each scanning mirror rotating along either the *x* or *y* axis. With the scan head inducing a 90° turn into the beam path, the incident light was directed through a telocentric lens pair, mounted in a 30 mm cage cube, and composed of an aspheric lens (*f* = 10 mm) and a plano-convex lens (*f* = 50 mm), leading to a 5× beam expansion. The incident light was then focused onto the sample by a long-working-distance IR 10× objective (Mitutoyo, NT46-403) generating SHG at 530 nm. Up to 650 mW of 1064 nm light could be delivered to the sample with this system with the use of the IR objective (compared with 140 mW with the upstream system). The SHG was detected in the epi-direction, collected through the incident objective and reflected through a filter set and onto a compact PMT module (Hamamatsu, H10722-10) by a dichroic mirror (Omega Optical, 580DCLP) centered around 532 nm and mounted in a rotatable kinematically controlled cage cube platform. The SHG signal was detected through a filter set composed of a KG3 (Thorlabs, FGS900) and 530 nm filter (Chroma, z532/10x). Bright-field images were also collected in the epi-direction using a module composed of an aspheric lens (*f* = 20 mm) and a CMOS camera (Thorlabs, DCC1645C), manually inserted when bright-field images were desired. Including the laser source, the total footprint of the microscope was 25 cm × 15 cm × 15 cm. The microscope was translated to the sample, at a height of 1.4 m, to perform SHG detection and centering measurements. The foundation of the microscope was a high-precision long-travel translation stage (Newport, M-IMS300V), and its electronics box (Newport, ESP 300, three-axis motion controller), capable of translating the laser pulse-compressor/output coupler, the microscope and the support structure to and from the sample between X-ray measurements, corresponding to approximately 20 cm of travel, with an absolute accuracy of 2 µm.

The electronics package was designed and constructed in collaboration with the Jonathan Amy Facility for Chemical Instrumentation at Purdue University (JAFCI). The electronics package integrated the electronics associated with the microscope, including the power supplies, control boards and data acquisition card (National Instruments), into a compact housing for easy mounting and transport, with a footprint of 46 cm × 61 cm × 31 cm. Data were acquired as photon counts using a gated multi-scalar card (Becker & Hickl, PMS-400a), controlled using a custom-designed *Labview* program, which was also written in collaboration with JAFCI. Data reconstruction and imaging were completed through *ImageJ* (NIH, 2011 ▶).

### 2.2. X-ray raster scan scheme   

XRD analysis and NLO images were acquired on all samples studied on 23-ID-B. Diffraction of kOR-T4L was acquired with a 5 µm-diameter X-ray beam, 5 × 5 µm cell size, 12.0 keV X-ray beam, with 1 s exposure times, a photon flux of 2.7 × 10^10^ photons s^−1^ (full unattenuated beam) and a detector distance of 300 mm. Diffraction of TsUCH37 was acquired with a 10 µm-diameter X-ray beam, a 10 × 10 µm cell, a photon flux of 1.3 × 10^10^ photons s^−1^ (10-fold attenuation) and detector distance of 300 mm. Diffraction of α-cellulose was acquired with a 10 µm-diameter X-ray beam, a 10 × 10 µm X-ray beam with a photon flux of 2.7 × 10^9^ photons s^−1^ (50-fold attenuation) and detector distance of 300 mm. The resulting NLO images and XRD raster measurements were compared using *ImageJ* and *JBluIce* (Hilgart *et al.*, 2011 ▶), which employs *DISTL* (Zhang *et al.*, 2006 ▶), to assess the degree of correlation of the sample position within the loop. The boundaries of the raster grids and raster cell sizes were defined using the software GUI *JBluIce* (Stepanov *et al.*, 2011*b* ▶). Bragg candidates, which estimate the number of well-ordered reflections, were generated for each X-ray diffraction image; they are shown color-coded in the figures as unsmoothed XRD raster images. The X-ray beam size was adjusted using a mini-beam collimator (Fischetti *et al.*, 2009 ▶).

## 3. Sample materials   

Phenylalanine hydroxylase from *Chromobacterium violaceum* (cPAH) was purified as a glutathione s-transferase (GST) fusion protein. The GST tag was cleaved with PreScission protease (GE Biosciences). For crystallization, cPAH was concentrated to 10 mg ml^−1^ in a solution of 5 m*M* HEPES, pH 7.4. Crystals of cPAH were obtained at ambient temperature utilizing hanging-drop vapor diffusion from solution 43 of Hampton Research’s PEG/Ion 2 screen [0.1 *M* Na-HEPES, pH 7.0, 0.01 *M* magnesium chloride hexahydrate, 0.005 *M* nickel (II) chloride hexahydrate and 15% *w*/*v* PEG 3350] with 8.3 m*M* hexammine cobalt (III) chloride and 8.3 m*M* guanidine hydrochloride as additives. Crystals were briefly soaked in 25% ethylene glycol and then flash-cooled in liquid nitrogen.

Crystals of human κ-opioid receptor in complex with an antagonist JDTic were obtained as described by Wu *et al.* (2012 ▶). Briefly, the human κ-opioid receptor sequence was modified by fusing T4 lysozyme (T4L) into intracellular loop 3 (Gly261–Arg263), performing N/C-terminal truncations (ΔGlu2Ala42, ΔArg359Val380) and introducing a single point mutation Ile135^3.29^Leu. The resulting construct kOR-T4L was expressed in baculovirus infected sf9 insect cells. Receptor was extracted from isolated membranes using dodecylmaltoside/cholesterol hemisuccinate detergent mixture, purified by metal-affinity chromatography, and concentrated to 40 mg ml^−1^. Lipidic cubic phase crystallization was performed as previously described (Caffrey & Cherezov, 2009 ▶; Cherezov *et al.*, 2004 ▶), by mixing protein solution with 10% cholesterol in monoolein at 2/3 protein solution/lipid ratio, and dispensing 50 nL protein laden LCP boluses overlaid with 800 nL precipitant solutions in a 96-well glass sandwich plate (Marienfeld) (Cherezov & Caffrey, 2003 ▶) using a NT8-LCP crystallization robot (Formulatrix). Crystals were obtained in 100 m*M* sodium citrate pH 5.8–6.4, 28–32% (*v*/*v*) PEG 400, 350–450 m*M* potassium nitrate, and were harvested directly from LCP matrix using MiTeGen micromounts and flash-cooled in liquid nitrogen.

The catalytic domain of *Trichinella spiralis* deubiquitinating enzyme UCH37 was expressed in *E. coli* as a GST-fused construct, purified on a glutathione-agarose column, complexed with ubiquitin vinyl methyl ester (UBVME), and subsequently purified by ion-exchange chromatography. Crystals of this complex, hereafter referred to simply as TsUCH37-UbVME complex, were grown by hanging-drop vapor diffusion in 3 *M* ammonium sulfate, 0.1 *M* bicine pH 9.0, and 2 m*M* l-glutathione (mixture of reduced and oxidized) over two days at room temperature.

The α-cellulose was prepared from pulpwood that underwent both the Kraft process and subsequent mercerization (Sixta *et al.*, 2004 ▶; Takai & Colvin, 1978 ▶).

A construct encoding the membrane domain of *E. coli* O157:H7 intimin was expressed, purified and crystallized as described previously (Fairman *et al.*, 2012 ▶). Briefly, Int208-449 was expressed in the outer membranes of *E. coli* BL21(DE3) cells, extracted with the detergent Elugent (Calbiochem), and purified by Ni-NTA affinity and anion-exchange chroma­tography using buffers containing dodecyl maltoside (Anatrace). Size-exclusion chromatography was used as a final purification step and served to exchange the detergent to lauryl dimethyl amine oxide (LDAO, Anatrace) using a buffer containing 50 m*M* Tris-HCl, pH 7.5, 200 m*M* NaCl, 0.01% NaN_3_ and 0.05% LDAO. The protein was concentrated to 20 mg ml^−1^, heptanetriol was added at 3% *w*/*v*, and the solution was mixed with monoolein at a 2/3 protein-to-lipid ratio. A Mosquito LCP robot (TTP Labtech) was used to dispense 100 nL protein–lipid droplets, overlaid with 750 nL well solutions. Intimin crystals grew from 100 m*M* sodium citrate, pH 4.5–5.5, 50–100 m*M* NaCl, 100–150 m*M* MgCl_2_ and 30–34% PEG 400. Crystals were mounted directly from the LCP mixture and flash-cooled in liquid nitrogen.

## 4. Results and discussion   

Data were acquired with both downstream and upstream versions of the NLO instrument, and schematic representations along with photographs of the beam paths are shown in Fig. 1 ▶.

**Figure 2:**
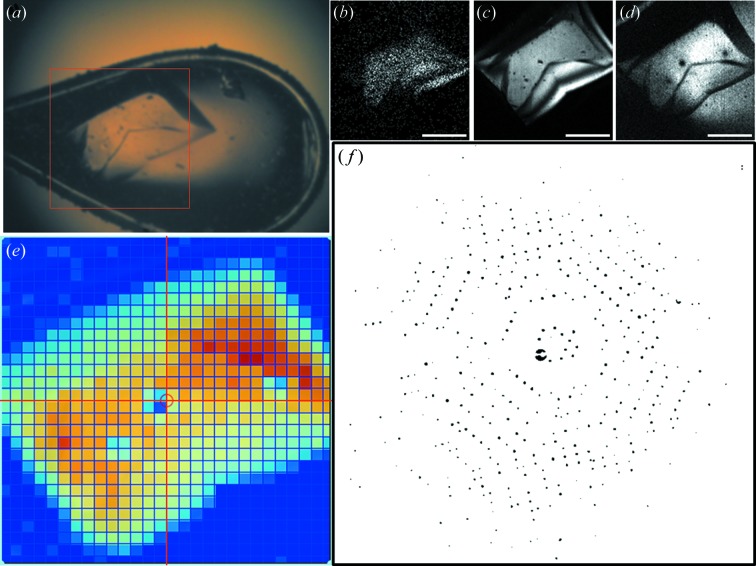
(*a*) Bright-field image of a *T. spiralis* UCH37 1-226/UbVME complex crystal (∼100 µm thick) and the corresponding (*b*) epi-SHG, (*c*) trans-SHG, (*d*) TPE-UVF and (*e*) X-ray raster scan within the 300 × 300 µm box. (*f*) X-ray diffraction of a representative 10 µm-diameter area from (*e*). X-ray energy: 12 keV; exposure time: 1 s; photon flux: 2.7 × 10^9^ photons s^−1^ (10-fold attenuation); detector distance: 300 mm; maximum theoretical resolution: 2.25 Å. The large difference in the epi- and trans-SHG signals is expected for thick samples owing to the difference in the forward and backward coherence length. The intensities of the two directions will approach equality as the sample thickness approaches the backwards coherence length (∼100 nm). Scale bars are 100 µm. (Three darkened spots, apparent in this figure, arose from separate X-ray ‘burn tests’ to assess X-ray damage, the results of which will be published in a future study.)

Fig. 2 ▶ (acquired *via* the upstream system) shows a large TsUCH37-UbVME crystal. Both the presence and position of the crystal can be independently confirmed with bright-field imaging (*a*), NLO microscopy and XRD measurements. Signal intensities of the corresponding epi-SHG (*b*), transmission-SHG (*c*) and TPE-UVF (*d*) were measured and processed in *ImageJ*. Although the crystal is visible using conventional optical imaging approaches, NLO microscopy produced substantial improvements in contrast compared with bright-field imaging. An X-ray diffraction raster was acquired (*e*) and a representative diffraction image is shown (*f*).

**Figure 3:**
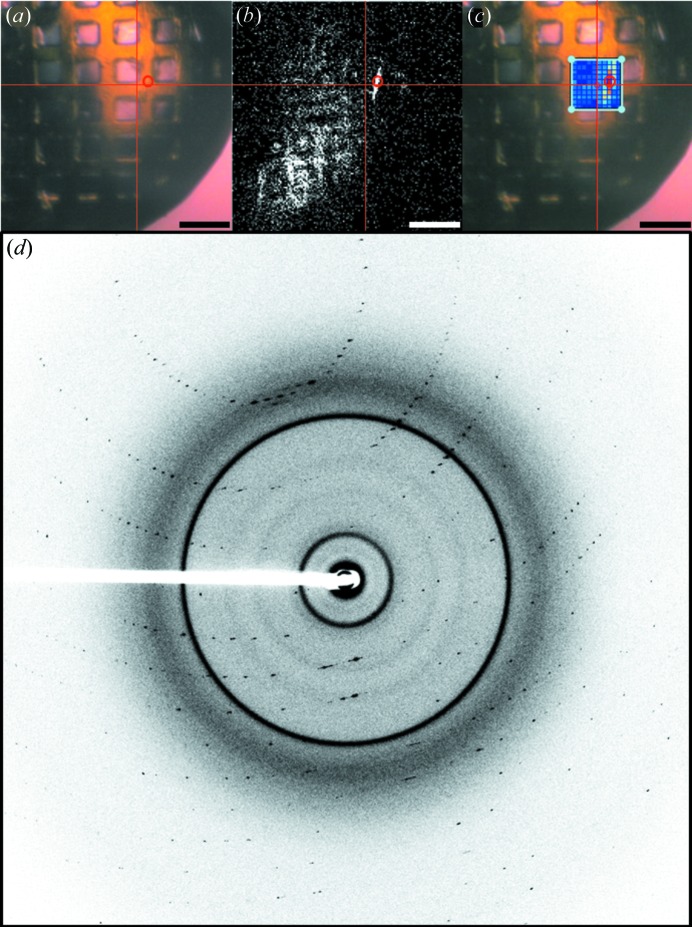
(*a*) Bright-field for an intimin protein crystal generated in LCP with corresponding (*b*) trans-SHG and (*c*) X-ray raster summary overlay showing corrected Bragg-like reflection counts. (*d*) X-ray diffraction of the 5 µm-diameter area corresponding to the red circles in each image, with X-ray energy 12.0 keV, exposure time 1 s, photon flux 2.7 × 10^10^ photons s^−1^ (unattenuated beam), sample-to-detector distance of 300 mm, resulting in a maximum theoretical resolution of 2.25 Å. Scale bars are 50 µm. Cross-hairs were added to (*a*) and (*b*) to assist in orienting the field of view with respect to the diffraction raster images.

Intimin protein crystals in LCP were examined using the upstream NLO system. In Fig. 3 ▶ the bright-field image is shown in (*a*), with the corresponding trans-SHG image (*b*), and X-ray raster acquired with a 5 × 5 µm beam, confirming the presence of a protein crystal (*c*), with the spot having greatest protein-like diffraction circled and the resulting diffraction pattern provided (*d*). All protein crystals identified by SHG and XRD were accurate for absolute position within the resolution of the 5 µm X-ray beam.

**Figure 4:**
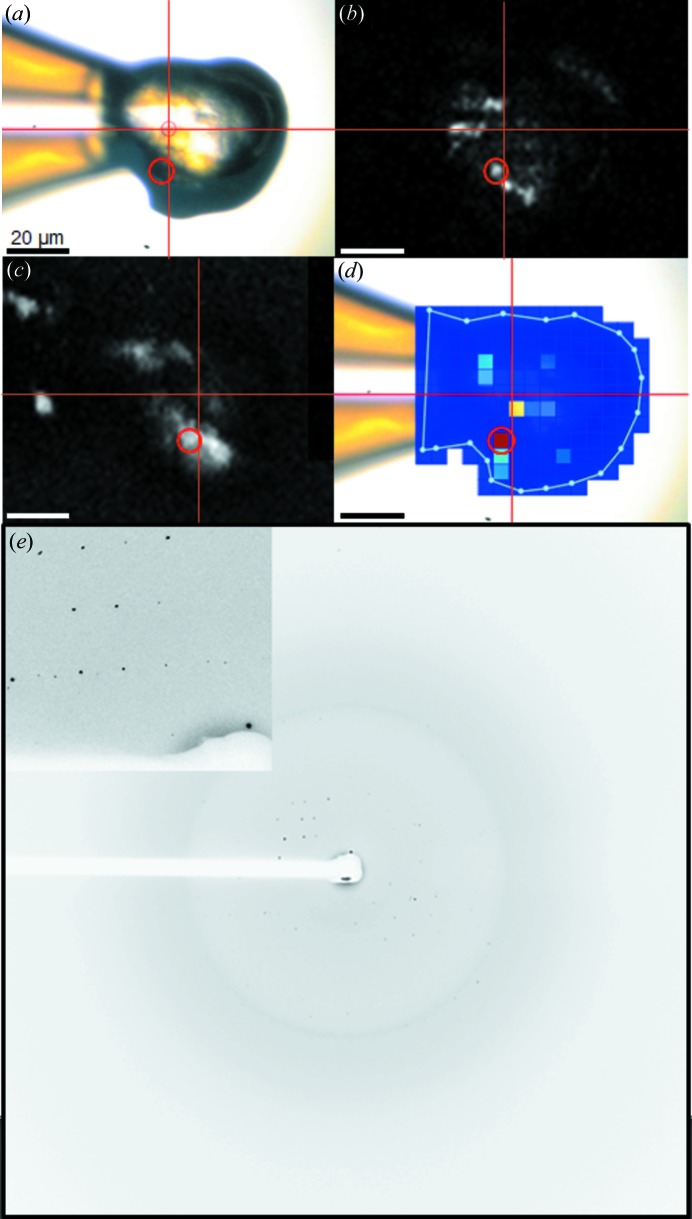
(*a*) Bright-field image of a membrane protein (human κ-opioid receptor complex) crystal in lipidic cubic phase and the corresponding (*b*) trans-SHG and (*c*) TPE-UVF, with (*d*) an X-ray raster summary overlay showing corrected Bragg-like reflection counts. (*e*) X-ray diffraction of the 5 µm-diameter area corresponding to the red circles in each image. X-ray energy: 12.0 keV; exposure time: 1 s; photon flux: 2.7 × 10^10^ photons s^−1^ (unattenuated beam); sample-to-detector distance: 300 mm; maximum theoretical resolution: 2.25 Å. Scale bars are 20 µm. Cross-hairs were added to (*b*) and (*c*) to assist in orienting the fields of view with respect to the bright-field and diffraction raster images.

In Fig. 4 ▶ (acquired *via* the upstream system) a bright-field image of a kOR-T4L crystal within frozen lipidic cubic phase is shown (*a*). As often arises with lipidic mesophase crystallizations, the looped droplets exhibited high optical scattering upon freezing that frustrated conventional bright-field imaging approaches for crystal positioning. Transmission SHG (*b*) and TPE-UVF (*c*) images were acquired, exhibiting localized areas (∼2–5 µm) of signal within the loop, suggesting the presence of a crystal. Crystals were confirmed *via* a 5 µm-diameter X-ray beam and 5 × 5 µm cell X-ray raster scan (*d*), in which several pixels exhibit weak, but detectable, diffraction with Bragg analysis consistent with the presence of a protein crystal. Diffraction patterns for the brightest spot are shown in Fig. 4(*e*) ▶. However, signal is observed in the trans-SHG and TPE-UVF images that does not correspond to areas of protein-like diffraction in the X-ray raster image. This signal discrepancy is tentatively attributed to protein crystals that are too small to produce Bragg peaks by XRD, or to the presence of other ordered materials arising in a false positive. False negatives for particular focal planes were also observed, in which analysis of the diffraction patterns obtained from the raster image indicates the presence of protein-like diffraction located in areas that did not exhibit substantial SHG or TPE-UVF due to the finite depth of field (∼25 µm). However, acquisition of multiple focal planes through samples has been observed to recover crystal locations more quantitatively (not shown).

In SHG measurements the possibility of false positives exists from other SHG-active structures. Most notably, some salts commonly used in crystallization screening can adopt non-centrosymmetric SHG-active lattices and produce bright SHG. Alternatively, noncrystalline structures exhibiting molecular ordering over distances significantly greater than the wavelength of light can also potentially produce false positives for SHG. An example of a false positive, from a noncentrosymmetric vanadate salt crystal, is shown in Fig. S1 of the supplementary information^1^ in which a cryo-loop containing a crystal grown in LCP was examined with the upstream NLO instrument, and yielded substantial signal in the epi- and transmission-SHG directions. X-ray raster scans suggested the presence of salt-like diffraction, in addition to ice diffraction, as there was ice present on the sample loop. Key signatures for an SHG-active salt were found to be bright epi-SHG and little to no detectable TPE-UVF. These salt crystal signatures can be exploited to reduce the likelihood of false positives. False positives can arise using TPE-UVF if there is protein aggregate located within the loop because TPE-UVF probes the presence of aromatic residues and is not crystal specific. Salt crystals and protein aggregates are common occurrences with protein crystal growth, generating false positives for SHG and TPE-UVF measurements, respectively. Fortunately, most simple salts adopt SHG-inactive centrosymmetric structures. Complementary use of these two techniques can significantly reduce the likelihood of false positives and false negatives.

**Figure 5:**
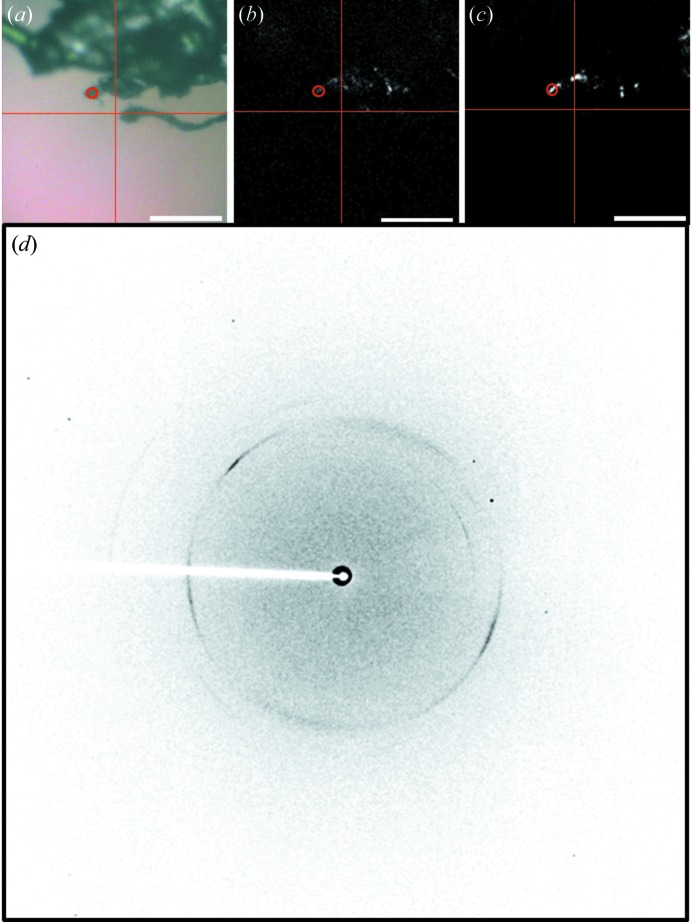
(*a*) Bright-field image of α-cellulose fibers and the corresponding (*b*) epi-SHG and (*c*) trans-SHG images, all 300 × 300 µm. (*d*) X-ray diffraction of a 10 µm-diameter area within the red circle of each image. X-ray energy: 12.0 keV; exposure time: 1 s; photon flux: 2.7 × 10^10^ photons s^−1^ (unattenuated beam); sample-to-detector distance: 300 mm; maximum theoretical resolution: 2.25 Å. Scale bars are 100 µm. Cross-hairs were added to (*b*) and (*c*) to assist in orienting the fields of view with respect to the bright-field image.

Combined NLO imaging and XRD was also applied to studies of α-cellulose, which exhibits fiber-like diffraction. NLO measurements performed on loop-mounted cellulose generated moderate S/N for multiple fibers within the sample loop (Fig. 5 ▶, acquired *via* the upstream system). Although fiber diffraction was evident from the cellulose samples, the DISTL algorithm used in raster scanning, which searches for discrete Bragg reflections or spots and not fiber diffraction, does not indicate these areas, but rather seems to show that no measurable sample is present. Manual inspection of the individual diffraction patterns was performed to discern the presence of fiber diffraction.

cPAH crystals ranging in size from 50 µm to 200 µm in length were imaged with both the downstream instrument with epi-only detection and X-ray raster scanning [Fig. S2 (supplementary information)]. The locations of intense protein-like Bragg diffraction typically agreed well with those of brightest epi-SHG for both large and small cPAH crystals (*e.g.* Fig. S2). However, departures between the two were also observed. Several explanations for the differences were considered. First, the presence of multiple crystalline domains within the crystal (*e.g.* from twinning) may cause the diffraction spot total to deviate from indicating optimal protein ordering. Second, inhomogeneous optical scattering of the incident or detected light can potentially impact the contrast through effects unrelated to the crystal SHG activity. However, bright-field images do not suggest substantial differences in optical transmissivity across the crystal that might have influenced contrast. Finally, NLO measurements probe a much narrower depth of field than X-ray diffraction, which is penetrating. If a particular crystal was not positioned within the depth of field of the beam-scanning NLO microscope, the SHG efficiency will be substantially reduced or entirely absent within the detection limits of the instrument. Despite the quantitative discrepancies, the presence of SHG signals above the background correlated with the areas of the crystal generating a detectable protein-like diffraction, providing preliminary confirmation of the ability of the downstream instrument to rapidly generate information for crystal position as a complement to X-ray raster scanning.

The polyimide loops (MiTeGen) were found to undergo noticeable deformation with less than 100 mW incident power using the downstream system, whereas the nylon loops were more robust, and were not damaged at these powers. No noticeable damage could be induced in either loop types using the upstream system during either SHG or TPE-UVF measurements (120 mW and 90 mW, respectively). Several mechanisms were considered for the observed laser-induced damage to the polyimide loops when measured with the downstream system. Previous studies suggest that damage from multi-photon absorption and plasma formation was found to be an important, if not dominant, mechanism for damage in biological NLO imaging (Sacconi *et al.*, 2006 ▶). However, those measurements were performed under conditions of tight focusing [high numerical aperture (NA)] and on live cells/tissues. However, alternative mechanisms may dominate in the present low-NA studies of purified protein crystals maintained under cryogenic conditions. Local heating was also considered as a possible damage mechanism, arising from either one- or two-photon absorption of the incident beam. The marked difference in damage susceptibilities between the upstream and downstream systems is consistent with this mechanism, differing notably in the use of a resonant 8 kHz scan mirror for the upstream system and a galvanometer-driven mirror operating at 200 Hz on the downstream system. Rapid beam-scanning using a resonant scanner combined with long-wavelength (>1 µm) incident light was shown previously to have no detectable effect on crystal diffraction quality using a variety of protein crystals, including myoglobin crystals containing heme groups exhibiting strong visible light absorption (Kissick *et al.*, 2013 ▶). Myoglobin was specifically chosen, as the color center was anticipated to be highly susceptible to light-induced perturbation (Banerjee *et al.*, 1969 ▶). However, no statistically significant structural changes to the lattice were observed in laser-exposed *versus* unexposed regions of single crystals (Kissick *et al.*, 2013 ▶).

The susceptibility for damage using the polyimide loops increased notably for TPE-UVF, as the optical transparency was substantially reduced at 530 nm. Whereas loop absorption is negligible at 1 µm for SHG, roughly 30% of the incident 530 nm light for TPE-UVF is absorbed by the standard yellow-tinted polyimide loop material (MiTeGen, http://www.mitegen.com/). By positioning the loop to avoid the outer turning points of the fast-scan mirror or blocking the beam at those locations, no noticeable damage could be induced in the polyimide loops during TPE-UVF imaging.

Both of the NLO imaging systems presented in this paper have strengths and limitations, and either could be utilized as a method for locating and centering protein crystals on a synchrotron beamline. With a small footprint and the ability to insert and remove the instrument, there is potential for a single design of the downstream instrument to be utilized on a variety of different beamlines. However, the time required for translating the entire microscope to and from the sample increases the total time for collecting SHG images and XRD of the protein. Indeed, the microscope positioning required substantially more time (∼2 min) than the sample imaging (∼40 s). Furthermore, the absolute accuracy of the translation stage (in this case, ±2 µm) can ultimately dictate the precision in crystal positioning. In addition, the downstream instrument did not have transmission-SHG detection capabilities. For protein crystals, detection in transmission provides substantial improvements in detection limits for weakly SHG-active proteins, as thickness greater than the crystals’ coherence lengths can decrease the overall SHG intensity in the epi direction (Boyd, 2009 ▶; Kestur *et al.*, 2012 ▶). The absence of transmission detection could potentially be remedied by introducing additional optics or integrating into existing optical paths.

The direct integration of the upstream system eliminated the need for a translation stage for inserting the microscope, as was used with the downstream system. This significantly reduced the time between imaging and XRD, which allowed for a marked improvement on throughput of data collection. The upstream system did still require the transmission detection optics to translate in and out for XRD collection in transmission, but epi-detected SHG can be performed concurrently with X-ray diffraction, with only a factor of three reduction in signal intensity with the mini-beam collimator in place. The positioning of the collection optics does not, however, require precise realignment allowing for a significant improvement on the translation time, as compared with the downstream instrument, where the entire microscope requires translation with high precision. The upstream system had some design trade-offs to accommodate the existing optical path, which in part accounted for the lower infrared (IR) throughput and available power in the upstream system. The biggest losses came from the incident objective in which 80% of the IR power was lost from reflections because it was not designed for IR incident light. Choosing optics with a more broadband anti-reflective coating (ARC) will significantly improve the power throughput. Testing performed in-house, with an IR-ARC objective, resulted in a doubling of the IR transmittance, corresponding to an anticipated four-fold improvement in signal at the sample (unpublished). The multiple imaging modes (SHG and TPE-UVF), as well as both epi and transmission detection, improves the ability of the upstream system to detect protein crystals that could otherwise be missed on the downstream system.

Based on these combined results, integrating a NLO microscope with a synchrotron XRD instrument complements stand-alone X-ray raster scanning for crystal centering in three key respects. First, it is expected to minimize radiation-induced sample damage compared with X-ray raster techniques for X-ray labile crystals or small crystals difficult to quickly detect at low X-ray flux (Kissick *et al.*, 2013 ▶). Second, NLO microscopy significantly increases the spatial resolution and reduces the total acquisition time for the determination of crystal location. For a large sample area (150 × 150 µm) scanned with a small beam size (5 × 5 µm), X-ray raster images for the protein crystals typically required approximately 30 min to acquire with a 1 s X-ray exposure time. For NLO measurements on identical samples, the acquisition time for the collection of each image was typically <10 s. The downstream NLO system allows 512 × 512 pixel images with 40 s acquisitions, and the upstream system allows 150 × 150 pixel images with 1 s acquisitions, which is roughly a >10^4^-fold reduction in the per-pixel acquisition time compared with the X-ray raster acquisition time per cell (∼3 s per pixel, corresponding to a 1 s exposure, with 2 s of dead-time between pixel acquisitions). The theoretical resolution of the objective was 1.6 µm with 2 µm measured spatial resolution. The downstream NLO system required a total time of 2.5 min for translation of the microscope from its resting position to the sample and then back to the resting position following NLO measurements, resulting in a total acquisition time for each sample of the order of 3 min, which is still significantly faster and of higher resolution compared with X-ray raster scan measurements performed on the same sample. In the upstream system, no dead-time was required for epi-detection (in fact, SHG imaging can be performed while acquiring diffraction measurements), and only a few seconds of translation time were required to raise and lower the collection optics in transmission. Third, for weakly diffracting systems where rapid automated diffraction scoring is challenging, NLO measurements may significantly increase the ability to locate protein crystals.

## 5. Conclusion   

Two different designs of integrated NLO instruments were constructed and characterized targeting applications for automated sample positioning. The systems were evaluated using protein crystals (TsUCH37-UbVME, kOR-T4L, cPAH, Intimin) and fibers (α-cellulose). Both NLO and XRD exhibited good agreement for crystal positioning, consistent with previous off-line measurements specifically targeting protein crystals (Kissick *et al.*, 2013 ▶). The integrated NLO and synchrotron XRD instrument was found to enable precise centering of α-cellulose samples for fiber diffraction without requiring the development of an application-specific analysis algorithm. The NLO instrument produced images with <10 s image acquisition times, compared with 3–60 min for X-ray rastering performed at much lower spatial resolution. By nature of the higher resolution of NLO image acquisition, the per-pixel raw data acquisition time was approximately five orders of magnitude faster than X-ray raster scanning. Once fully developed, NLO imaging may serve to identify regions of interest for targeted X-ray scanning, or ultimately serve as the sole or primary method for precise automated crystal positioning, such that all of the X-rays striking the crystal are dedicated to structure elucidation.

Despite these successes, a relatively small variety of crystals were used to characterize the instruments in this initial study. Further studies on a greater diversity of protein crystals will help define the scope of use for NLO methods in automated centering. Additionally, the present study focused exclusively on the hardware for visualization, and not on subsequent algorithms for image analysis and automated crystal positioning. Higher contrast afforded by NLO imaging has the potential to significantly improve the reliability of such algorithms if the combined techniques of SHG and TPE-UVF provide sufficient protein crystal coverage for general-purpose use.

These studies provided a foundation for future efforts combining NLO measurements with synchrotron X-ray diffraction. The data presented here support the use of the NLO microscopy for automated or manual crystal centering prior to or *in lieu* of raster scanning. Potential scope of use where all optical crystal positioning would be preferred includes the analysis of smaller crystals (<5 µm), where the low crystal volume may present challenges for rapid crystal positioning by X-ray raster scanning. SHG also enables positioning of fibrous material exhibiting fiber diffraction, such as cellulose, collagen, chitin *etc*. Further potential applications include defect studies, X-ray damage studies and studies of active pharmaceutical ingredients.

## Supplementary Material

Supporting information file. DOI: 10.1107/S0909049513007942/wa5051sup1.pdf

## Footnotes

1 Supplementary data for this paper are available from the IUCr electronic archives (Reference: WA5051). Services for accessing these data are described at the back of the journal.
