# Supplementary material for: Integrated nonlinear optical imaging microscope for on-axis crystal detection and centering at a synchrotron beamline
Source: J Synchrotron Radiat. 2013 May 3;20(Pt 4):531–40. doi: 10.1107/S0909049513007942 (PMC3682636; doi:10.1107/S0909049513007942)
Supplement: Supplementary file 1 [file s-20-00531-sup1.pdf]

WA5051 Supplementary Material
Figure S1

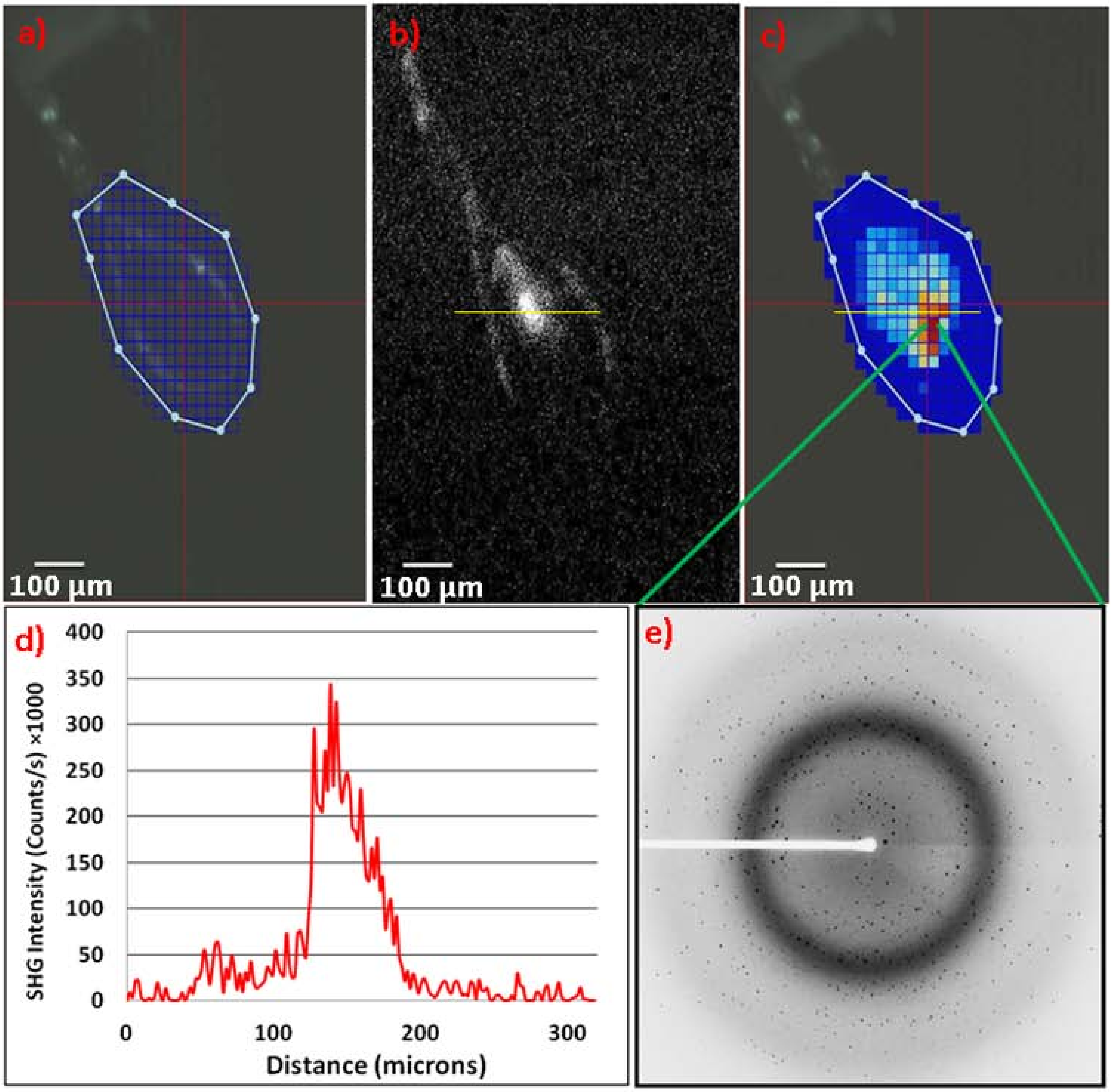

Figure S1. Centering measurements of a cPAH crystal with the downstream system, showing a) brightfield b) SHG image, and c) X-ray raster scan image, acquired with a 20 µm X-ray beam. Comparison of these two measurements showed areas of overlap in both the SHG image and the X-ray raster, as indicated by the d) SHG line trace analysis (yellow lines in (b) and (c)). Strong protein-like diffraction pattern corresponding to regions of strong SHG signal, as shown in e). There is a slight disagreement between the SHG image and raster image due to differences in focal planes.

Supplementary Material WA5051
Figure S2

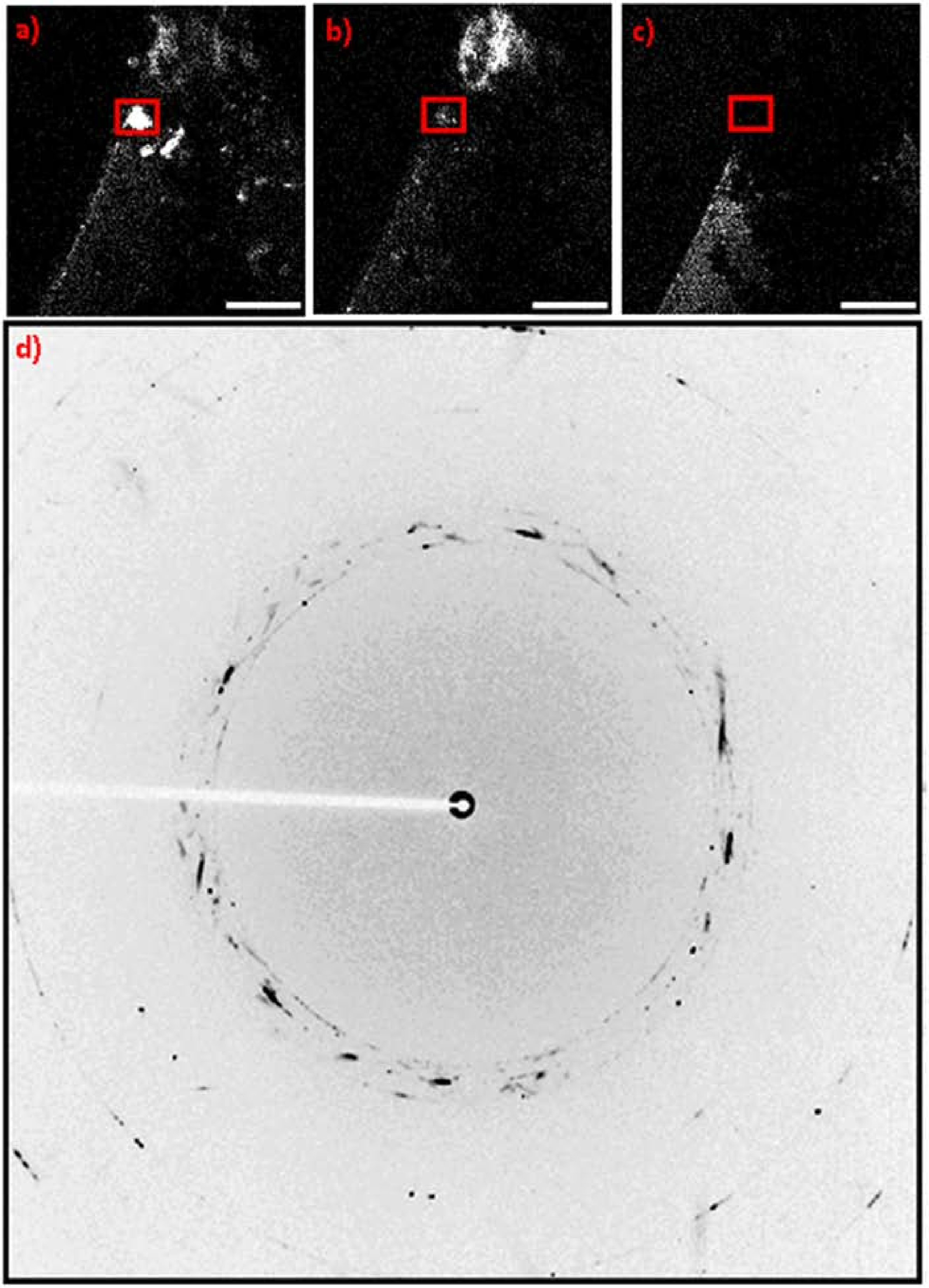

Figure S2. Images of an LCP crystal prepared in the presence of vanadate salts was imaged with the upstream system, a) epi-SHG, b) trans-SHG, and c) TPE-UVF. Strong SHG signal was detected in the epi direction from the area surrounded by the red box. Weak SHG signal was detected in the trans direction and no TPE-UVF signal was detected. A diffraction pattern d) indicating salt diffraction from vanadate salt, in addition to ice diffraction, obtained from a spot in the red box. The outer edges are 2.25 Å and the corners are 1.71 Å. The scale bars are 100 µm.
